# Strain Effects on the Electronic and Optical Properties of Kesterite Cu_2_ZnGeX_4_ (X = S, Se): First-Principles Study

**DOI:** 10.3390/nano11102692

**Published:** 2021-10-13

**Authors:** Jawad El Hamdaoui, Mohamed El-Yadri, Mohamed Farkous, Mohamed Kria, Maykel Courel, Miguel Ojeda, Laura M. Pérez, Anton Tiutiunnyk, David Laroze, El Mustapha Feddi

**Affiliations:** 1 Group of Optoelectronic of Semiconductors and Nanomaterials, ENSAM, Mohammed V University in Rabat, Rabat 10100, Morocco; jawadelhamdaoui11@gmail.com (J.E.H.); md.yadri@gmail.com (M.E.-Y.); mohammedfarkous@gmail.com (M.F.); mohamedkria1@gmail.com (M.K.); e.feddi@um5s.net.ma (E.M.F.); 2 Centro Universitario de los Valles (CUValles), Universidad de Guadalajara, Ameca 46600, Mexico; maykelcourel@gmail.com (M.C.); miguelojedama@gmail.com (M.O.); 3 Instituto de Alta Investigación, Universidad de Tarapacá, Casilla 7 D, Arica 1000000, Chile; tyutyunnyk.a.m@academicos.uta.cl (A.T.); dlarozen@uta.cl (D.L.)

**Keywords:** kesterite, CZGS, CZGSe, strain engineering, optical properties

## Abstract

Following the chronological stages of calculations imposed by the WIEN2K code, we have performed a series of density functional theory calculations, from which we were able to study the effect of strain on the kesterite structures for two quaternary semiconductor compounds Cu${}_{2}$ZnGeS${}_{4}$ and Cu${}_{2}$ZnGeSe${}_{4}$. Remarkable changes were found in the electronic and optical properties of these two materials during the application of biaxial strain. Indeed, the band gap energy of both materials decreases from the equilibrium state, and the applied strain is more pronounced. The main optical features are also related to the applied strain. Notably, we found that the energies of the peaks present in the dielectric function spectra are slightly shifted towards low energies with strain, leading to significant refraction and extinction index responses. The obtained results can be used to reinforce the candidature of Cu${}_{2}$ZnGeX${}_{4}$(X = S, Se) in the field of photovoltaic devices.

## 1. Introduction

The family of copper-based quaternary semiconductor compounds Cu${}_{2}$Zn(Sn, Ge)S${}_{4}$ has attracted much attention. They have been intensively investigated in order to introduce them to various applications: topological insulator [1,2], solar cells [3,4], thermoelectric [5,6] and non-linear optics [7]. Their p-type conductivity, the magnitude of their direct band gap and their absorption coefficient, superior to 10${}^{4}$ cm${}^{-1}$ [8,9,10,11,12], make them an excellent light absorbers, which are required in PV technology to improve the solar cells’ performance [13,14].

With respect to the protection of the environment, these compounds constitute a promising alternative to the rare and toxic materials generally used in PV devices, such as CdTe, GaAs or CIGS. Indeed, Cu${}_{2}$Zn(Sn, Ge)S${}_{4}$ absorbers are earth-abundant, non-toxic and low-cost. Therefore, their mastery constitutes a challenge for both researchers and industrials in order to manufacture environmentally friendly and low-cost cells with a high performance. Several theoretical and experimental studies have contributed to understanding the fundamental aspects of their physical properties. A brief literature overview shows that the majority of CZTS compounds crystalize as orthorhombic with space group Pmn2${}_{1}$ as tetragonal with space group I$\bar{4}$2m and I$\bar{4}$, which are related to the wurtzite or zinc blend, respectively [15]. They are known to have a direct band gap of around 1.5–1.97 eV [7,16], with a favorable optical absorption coefficient of about $10^{4}$–$10^{5}$ cm${}^{-1}$ [17,18,19,20,21,22,23].

Strictly speaking, the changes in the composition of copper-based quaternary chalcogenide semiconductors have improved their suitability as an absorber layer in photovoltaic cells. The substitution of tin (Sn) atoms by germanium (Ge) one can increase the optical band gap, which means that it can be combined with a low band-gap cell in a tandem structure that could convert a large portion of sunlight into electrical energy [24,25]. In these conditions, Cu-based quaternary chalcogenide semiconductors Cu${}_{2}$ZnGeX${}_{4}$ (X = S, Se), designed as CZGX (X = S or Se), are considered better photo-absorber materials in photovoltaic applications, as compared to CZTS, CIGS or CdTe.

Some experimental studies have investigated these materials: Parasyuk et al. [26] investigated the formation of the Cu${}_{2}$ZnGeS${}_{4}$ phase. Using X-ray powder diffraction, the authors showed that this compound crystallizes in the stannite phase with space group (I$\bar{4}$2m) with the lattice parameters a = 0.5606 nm, and c = 1.104 nm. Investigations into the structural and optical properties of CZGX (X = S or Se) thin films synthesized by chemical spray pyrolysis were realized by B. Dhruba et al. [27]. The authors investigated the morphology, structure and optical properties of these thin films. The band gap energy was estimated as 1.88–1.93 eV for post-sulfurized Cu${}_{2}$ZnGeS${}_{4}$ (CZGS) films and 1.40–1.43 eV for post-selenized Cu${}_{2}$ZnGeSe${}_{4}$ (CZGSe) films by UV-Visible absorption measurements. Recently, it was demonstrated that the inclusion of Ge in the synthesis of Cu${}_{2}$ZnGe(S, Se)${}_{4}$ absorbers for kesterite is an eventual way to increase the solar cells’ efficiency. To understand the mechanisms by which Ge improves the kesterite solar cell properties, Giraldo et al. [28] show that control of the position and thickness of the thin layer of Ge can influence the crystallization of kesterite thin films prepared in a sequential process. Indeed, Ge induces fundamental changes in the formation mechanism of the kesterite absorber. Using this structure, the authors show that conversion efficiencies of up to 11.8% can be obtained. El Radaf et al. [29] decribed the preparation of good-quality copper zinc germanium sulfide Cu${}_{2}$ZnGeSe${}_{4}$ (CZGSe) thin films and heterojunctions using spray pyrolisis. The X-ray analysis confirmed the crystalization of CZGSe in tetragonal structures. The authors demonstrated that these materials exhibit a direct band gap energy ranging between 1.52 and 1.27 eV, and the optoelectronic and nonlinear optical parameters are very sensitive to the increasing thickness. Recently, Courel et al. [30] described the preparation of Cu${}_{2}$ZnGeS${}_{4}$ thin films by thermal evaporation technique, starting from CuS, GeS and ZnS precursors and a post-deposition thermal processing. Based on the different characterization results, the authors concluded that this material has good physical properties for photovoltaic applications.

From a theoretical perspective, the stability and band diagrams of CZGX (X = S or Se) were studied by Chen et al. and Zang et al. [31,32]. They showed the existence of three fundamental species, i.e., kesterite, stannite and primitive mixed CuAu-like structure. Density functional theory (DFT) calculations were accomplished in order to understand the physical properties of the Kesterite using germanium. The electronic and optical properties of Cu${}_{2}$ZnGeS${}_{4}$ were calculated using the modified Becke–Johnson exchange correlation potential (mBJ) and generalized gradient approximation (GGA). According to another work, the energy gaps calculated using GGA and mBJ were 0.50 eV (GGA) and 1.21 eV (mBJ) [33]. The structural, electronic and optical properties of both Cu${}_{2}$ZnGe(S)${}_{4}$ and Cu${}_{2}$ZnGe(Se)${}_{4}$ semiconductor materials in the kesterite and stannite phase were investigated by Gupta et al. [34]. They found a band gap energy about 1.15 eV and 0.64 eV for Cu${}_{2}$ZnGeS${}_{4}$ and Cu${}_{2}$ZnGeSe${}_{4}$ in the kesterite structure, respectively, using (mBJ). However, both GGA and mBJ underestimated the value of the band gap energy. It has been reported that the use of mBJ combined with Hubbard potential gives a reasonable result that could be compared with the experimental data. M. Mesbahi et al. obtained a band gap of 2 eV with TB-mBJ [35]. It is usually convenient to introduce a corrective potential for accurate calculations of electronic structures, such as DFT+U correction. Indeed, The Hubbard term “U” is a semiempirical value that could be added to the standard DFT calculations, and it is applicable to all open-shell orbitals such as d or f orbitals. This approach has been demonstrated to be as reliable as the other methods, but with a critical advantage of its considerably lower computational cost [36].

To our knowledge, there is no study concerning the effect of the strain on these kind of structures CZGX(X = S, S${}_{e}$). In the present study, using the first principal calculations, we propose to determine the changes in the band structure and optoelectronic properties induced by strain. To do this, we have proceeded as follows: In Section 2, we provide the basics of the parameters, which are necessary to perform the DFT calculations. The structural, electronic and optical properties under biaxial strain are discussed in Section 3. Section 4 summarizes the main achievements of this work and the conclusion.

## 2. Details of Calculations

In this section, we will exhibit the essential data of the input file to perform DFT calculations for both CZGS and CZGSe kesterite structure using the full-potential linearized augmented plane-wave (FP-LAPW) approach, which is currently one of the most accurate implementations of the Kohn–Sham DFT, and well adapted for the crystalline systems implemented in WIEN2k code [37,38].

First, the exchange and correlation potential was treated by the Tran–Blaha-modified Becke–Johnson potential combined with the Hubbard potential U [39]. We used Hubbard’s potential to properly manipulate the electronic behavior of d-orbital atoms, which were Cu ([Ar] 4s${}^{1}$ 3d${}^{10}$), Zn ([Ar] 4s${}^{2}$ 3d${}^{10}$) and Ge ([Ar] 4s${}^{2}$ 3d${}^{10}$ 4p${}^{2}$ ). After testing U values between 0.3 Ry and 0.52 Ry, the final U value used in our calculations was set as 0.48 Ry. To achieve the energy eigenvalue convergence, we used sufficiently large muffin-tin radii to avoid their overlapping. The cut-off parameters used for plane wave were $R_{mt}*K_{max}=9$ (where $R_{mt}$ and $K_{max}$ are the plane wave radius and the maximum modulus for the reciprocal lattice vector, respectively). The core cut-off energy, which defines the separation of core and valence states, is set to −6 Ry, and the number of K-points in the whole brilllouin zone was fixed at 1000. The lattice parameter c/a and the volume of structure of both two considered kesterite compounds were optimized from the experimental parameters using the 2D optimize package [40] with the PBEsol approximation [41].

To calculate the optical properties, the following dielectric function formula $\varepsilon \left(\omega \right)=\varepsilon_{re}\left(\omega \right)+i\varepsilon_{im}\left(\omega \right)$ was used, where $\varepsilon_{re}$ stands for its real part, obtained using the Kramer–kroning transformation [40], and $\varepsilon_{im}$ denotes its imaginary part, which can be given by the momentum matrix element between the occupied and unoccupied states according to the following equation:

(1) $$\varepsilon_{im}\left(\omega \right)=\frac{e^{2}\hbar }{\pi m^{2}\omega }\sum_{v,c}\int_{BZ}{\left|\langle ck\left|e\nabla \right|vk\rangle \right|}^{2}\delta \left(\omega_{ck}\left(k\right)-\omega \right)d^{3}k$$

where *c* and *v* are the conduction and valence of Kohn–Sham states, respectively.

(2) $$\varepsilon_{re}\left(\omega \right)=1+2/\pi \int_{0}^{\infty }\frac{\varepsilon_{i}\left(\omega^{\prime }\right)}{{\omega^{\prime }}^{2}-\omega^{2}}\omega^{\prime }d\omega^{\prime }$$

Other optical properties, such as the refraction index n(ω), extinction coefficient k(ω) and absorption coefficient α(ω), can be calculated from dielectric function parts as follows:

(3) $$n\left(\omega \right)=\frac{1}{2}{\left[{\left(\varepsilon_{re}^{2}\left(\omega \right)+\varepsilon_{im}^{2}\left(\omega \right)\right)}^{1/2}+\varepsilon_{re}\left(\omega \right)\right]}^{1/2}$$

(4) $$k\left(\omega \right)=\frac{1}{2}{\left[{\left(\varepsilon_{re}^{2}\left(\omega \right)+\varepsilon_{im}^{2}\left(\omega \right)\right)}^{1/2}-\varepsilon_{re}\left(\omega \right)\right]}^{1/2}$$

(5) $$\alpha \left(\omega \right)=\frac{\sqrt{2\omega }}{c}{\left[{\left(\varepsilon_{re}^{2}\left(\omega \right)+\varepsilon_{im}^{2}\left(\omega \right)\right)}^{1/2}-\varepsilon_{re}\left(\omega \right)\right]}^{1/2}$$

For the induced strain, a desired rate $\varepsilon_{b}$ of biaxial stress was applied either by stretching the lattice constant *a* (tensile strain to $a_{+}=a+a\varepsilon_{b}$) or reducing it (compressive strain $a_{-}=a-a\varepsilon_{b}$). Finally, for each considered case of strain, we adjusted the c parameter and we promoted the relaxation of the structure [42].

## 3. Results and Discussion

### 3.1. Structural Properties

**Figure 1:**
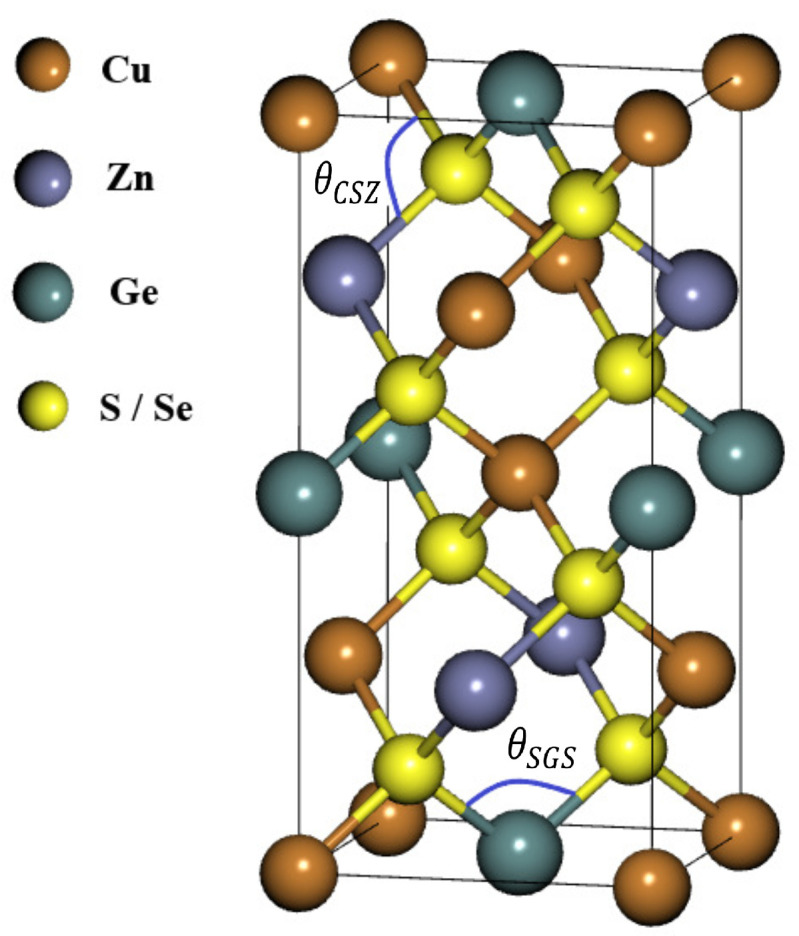
Schematic representation of Cu${}_{2}$ZnSnS${}_{4}$ and Cu${}_{2}$ZnSnSe${}_{4}$ in their kesterite structure.

**Figure 2:**
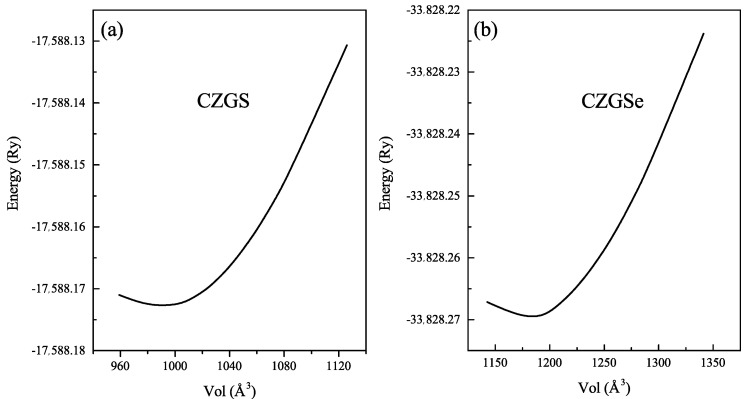
Total energy as a function of volume of (**a**) CZGS and (**b**) CZGSe in kesterite structure.

Before starting the analysis of the results, we draw attention to the stable crystal structure of CZGX (X = S, Se) presented in Figure 1 [43]. The relaxed lattice parameters of CZGX (X = S, Se) were acquired by fitting the total energy as a function of the volume of the unit cell using Murghan’s equation of state [44]. The Figure 2 presents the fitted data of total energy to unit cell volume. Thus, the optimization process leads to the following equilibrium parameters (a, c) (5.28, 10.515 Å) for CZGS and (5.58, 11.11 Å) for CZGSe, which are consistent with other theoretical and experimental works [45]. Table 1 compares our obtained calculations of lattice constants, band gap energy, static dielectric constant and the refraction index with some theoretical and experimental works from the literature.

### 3.2. Electronic Properties

**Figure 3:**
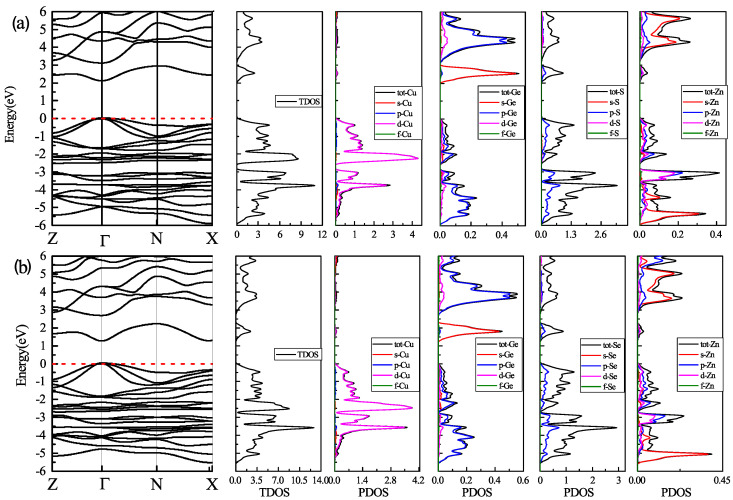
Schematic band structure, total and partial density of state mapped out from DFT/GGA calculations using the mBJ + U potential: (**a**) for CZGS and (**b**) for CZGSe. The dashed line refers to the Fermi level.

First, we investigated the band gap energy for both two kesterite compounds with mBJ + U calculations. Figure 3 gives an overview of the band structure a) for CZGS material and b) for CZGSe. As can be seen from this figure, the valence band maximum (VBM) and the conduction band minimum (CBM) are located at the Γ point of the first Brillouin zone for both materials, which means that the two materials have a direct band gap. We note that the addition of the Hubbard potential leads similar behaviour of the band gap, as reported by [45]. The values of the band gap energy were about 2.05 eV and 1.26 eV for CZGS and CZGSe, respectively. These values are close to the experimental measurments reported by Reference [27]. For more details on the origin of the electronic band structure, the corresponding partial and total density of states are also presented in Figure 3. The band within the range from −6 to −4 eV in the valence band is a hybridization between the s-orbital of Zn and p-orbitals of S/Se and Ge; meanwhile, the bands near the Fermi level are dominated by Cu-d orbitals with a little contribution from p-orbitals of S/Se and Ge. On the other side of the band structure, the bottom of the conduction band is essentially formed from the s-Ge and p-S orbitals; the rest of the conduction band is a coupling between p-Ge and s-Zn orbitals.

**Figure 4:**
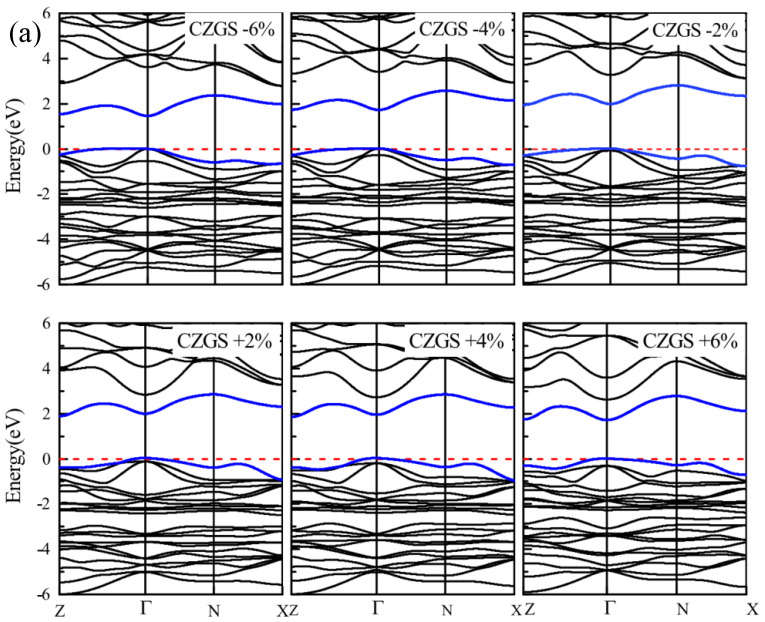

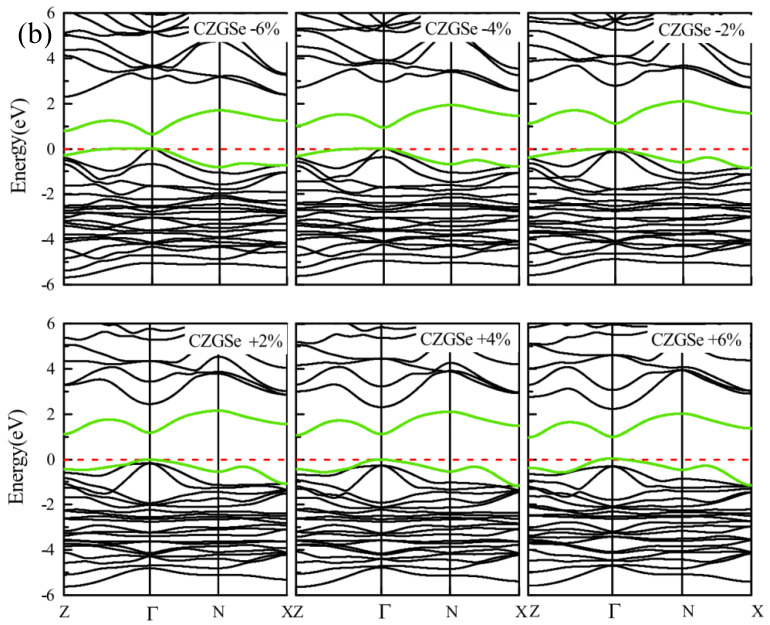
Variation of band structure with lattice parameter deformation (**a**) for CZGS and (**b**) for CZGSe.

**Figure 5:**
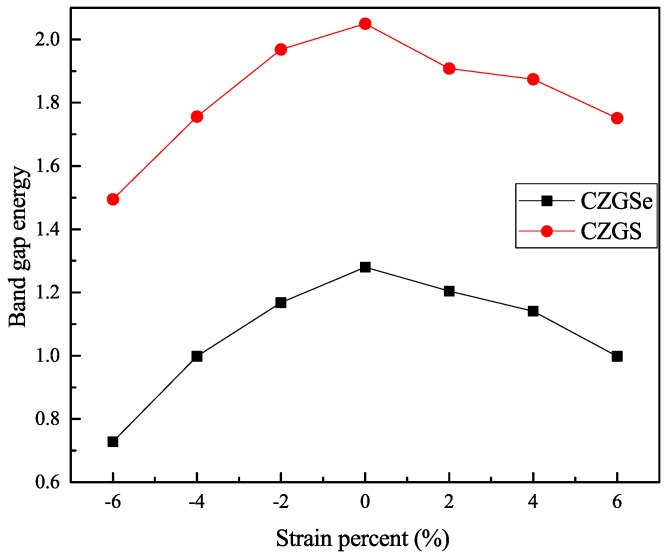
The variation of band gap energy under tensil and compressive biaxial strains.

By the application of strain on the structure, the gap value is completely modified; the band structure of both CZGS and CZGSe with different rates of applied strain is presented in Figure 4. The percentage range of the biaxial strain was chosen as between −6% and +6% by step of 2%, where the 0% represents the equilibrium states, as shown in Figure 3 and the negative (positive) represents compressive (tensile) strain. In all cases, no changes were observed on the nature of the band gap; whatever the intensity of the applied strain, the materials always present a direct band gap at Gamma point. However, the difference appears on the energy band gap value in comparison to the equilibrium state. To highlight this difference, we propose to illustrate, in Figure 5, the variation in the band gap energy versus the biaxial strain. As we can see, the bandgap energy is more sensitive to compressive strain than the tensile one. This is due to the strong interaction between atoms, due to the shrinkage of the lattice parameter in the case of compressive strain. The gap value varied from 2.05 to 1.495 eV and from 1.28 to 0.728 eV for CZGS and CZGSe, respectively, while the bandgap under tensile decreased to 1.751 and 0.998 for CZGS and CZGSe, respectively.

**Figure 6:**
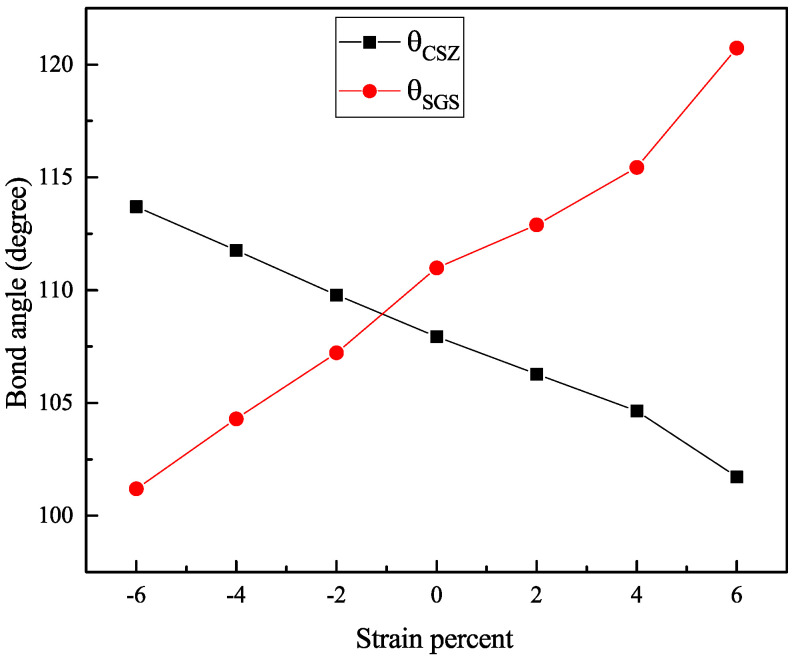
Variation of bond angles $\theta_{SGS}$ and $\theta_{CSZ}$ with strain.

We found that the variation in the gaps under strain is mainly due to the shift in the conduction band and the valence band in both materials. These changes in the electronic properties are related to the geometric modification of both structures, induced by the lattice distortion that manifests in the bond angle of the structure. To inspect the bond angle change in the kesterite structure, we chose two angles: the first is ∠SGeS angle, named $\theta_{SGS}$, which represents the change in the bond angle in the perpendicular plane (see Figure 1); the second is ∠CuSZn, named $\theta_{CSZ}$, representing the parallel bond angle (See Figure 1). Figure 6 presents the variation in $\theta_{SGS}$ and $\theta_{CSZ}$ with strain. We note that CZGS and CZGSe exhibit the same behaviour. For clarity, we show the variation in the CZGS bond angle alone. It can be seen that $\theta_{SGS}$ significantly expands with the strain caused by stretching the lattice parameters a and b. $\theta_{SGS}$ expansion is accompanied by a reduction in $\theta_{CSZ}$, affected by the lessening of the lattice parameter c. For the case of compressive strain, the lessening of $\theta_{CSZ}$ can provide an explanation for the reduction in band gap energy; meanwhile, the change in $\theta_{SGS}$ is responsible for lowering the band gap energy in tensile strain. 

To obtain an idea of the band discontinuiy at the interface, in the case where we consider CZGS/CZGSe heterostructure, we used the relative positions of valence and conduction bands for both materials. These values are extracted from the the band diagrams given in Figure 3. The band offsets are estimated following the same formula given in reference [48]

(6) $$\Delta E_{v}=E_{v}\left(CZGS\right)-E_{v}\left(CZGSe\right)\;\;\;\mathrm{and}\;\;\;\Delta E_{c}=E_{c}\left(CZGS\right)-E_{c}\left(CZGSe\right)$$

**Figure 7:**
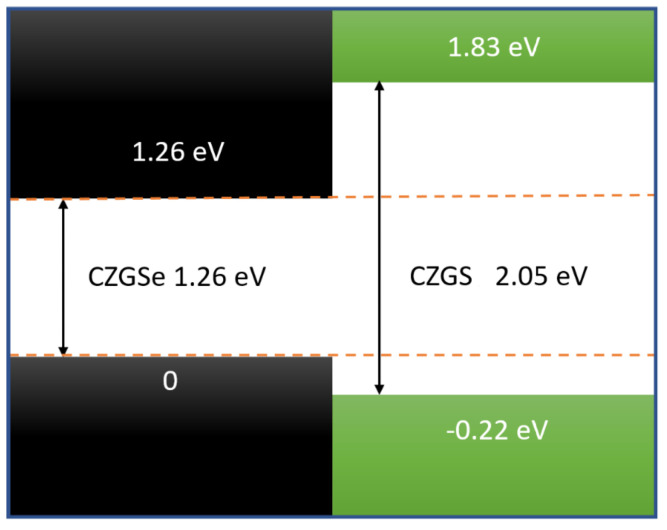
Schematic view of the band discontinuity of CZGS/CZGSe heterostructure.

This band dicontuinity of CZGS and CZGSe is presented in Figure 7. As we can see, the bandgap of CZGSe is completely contained in CZGS bandgap, which makes the alignment between both materials a straddling type (type I), with a valence and conduction band offset of 0.22 and 0.77 eV, respectively. The band offset of CZGS/Se is larger than the reported bands’ offset of the well-studied kesterite Cu${}_{2}$ZnSnS/Se 0.35 and 0.15 eV [45], which is a result of the substitution of Sn by Ge, resulting in an increased bandgap of kesterite material. The minimum of the conduction band is the antibonding state s of Ge, while the maximum of the valence band is the bonding d of Cu.

### 3.3. Optical Properties

**Figure 8:**
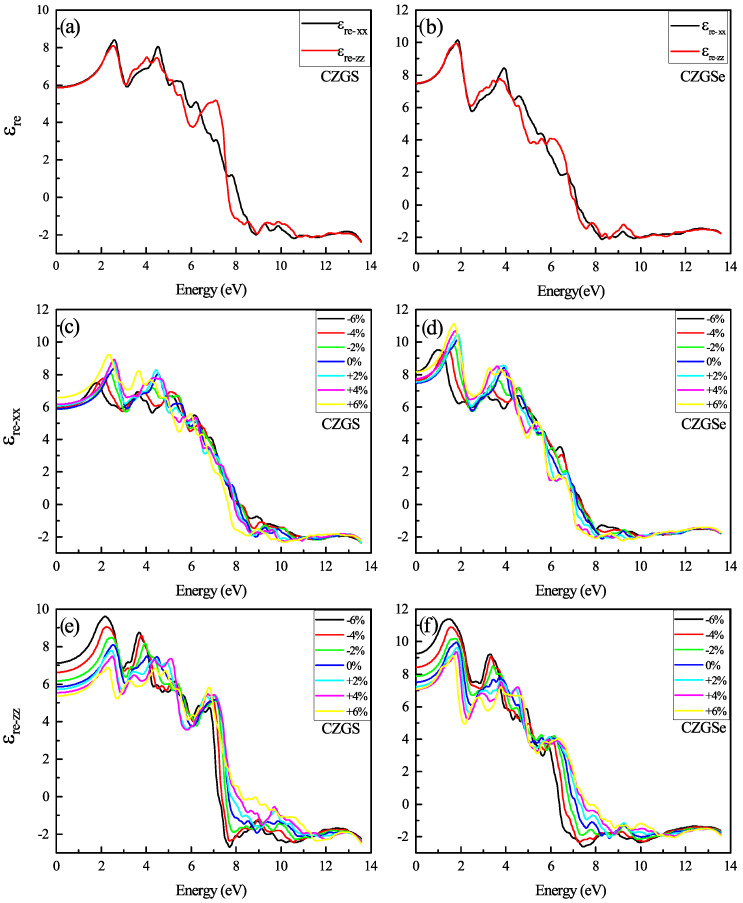
The variation of the real part of the dielectric function spectra as a function of the incident photon energy for both materials CZGS and CZGSe: (**a**,**b**) are the unstrained cases, (**c**–**f**) are the strained cases. $\varepsilon_{re-xx}$ and $\varepsilon_{re-zz}$ are the real parts of the dielectric function for light polarization along XX and ZZ respectively.

**Figure 9:**
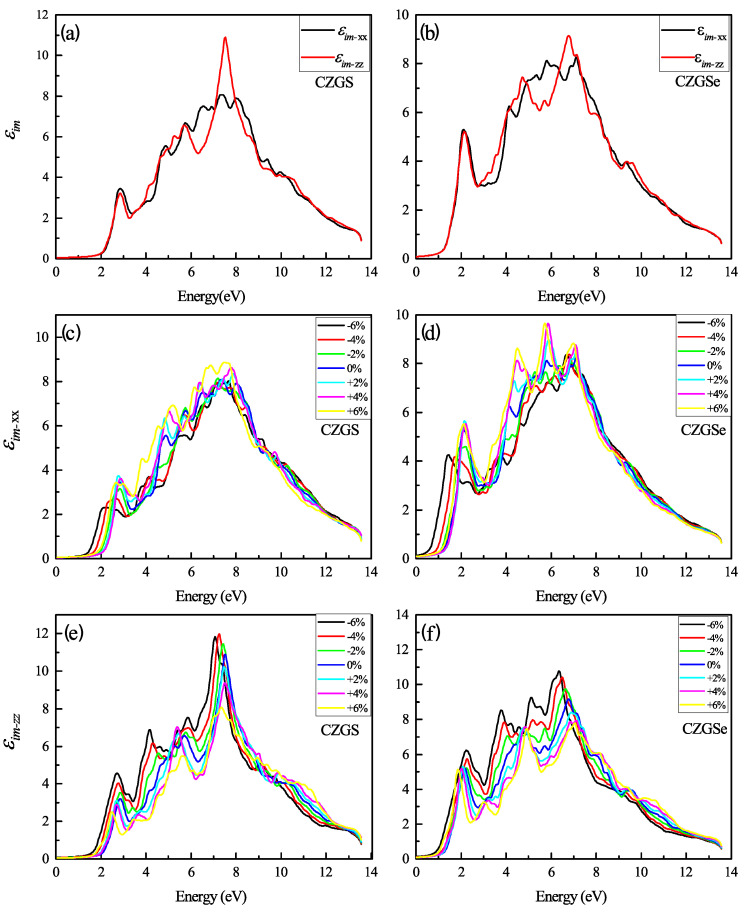
The variation of the imaginary part of the dielectric function spectra as a function of the incident photon energy for both materials CZGS and CZGSe: (**a**,**b**) are the unstrained cases, (**c**–**f**) are the strained cases. $\varepsilon_{im-xx}$ and $\varepsilon_{im-zz}$ are the imaginary parts of the dielectric function for light polarization along XX and ZZ directions respectively.

The optical properties of quaternaries CZGS/Se in the tetragonal structure could be characterized by two directions of light polarization ( transverse and longitudinal direction). The first analysis of optical characteristics is devoted to the fluctuations in the dielectric function as a function of frequency. In Figure 8a,b, the energy-dependent real part of the dielectric function is shown for both CZGS and CZGSe kesterite materials. It is clear from the Figure 8, that the real part of CZGSe is higher than that of the CZGS. This means that selenium-based kesterite exhibits a higher light dispersion. On the other hand, the dielectric imaginary part is illustrated in Figure 9a,b for transversal and longitudinal polarization. The spectra show a difference in the intensity of the first peak, which corresponds to the first interband transition between the VBM and CBM. This difference is caused by the fact that CZGSe has a smaller bandgap energy. The absorption is strongly correlated to the imaginary part of the dielectric function, where its maximum is observed in the range of 6–8 eV.

In Figure 8c–f the real part of the dielectric function spectra of both CZGS and CZGSe are represented by both CZGS and CZGSe for different biaxial strain rates. It can be shown that the first peak in the spectra shows an energy threshold that corresponds to the value of the band gap energy. Therefore, it represents the first direct optical transition between the VBM and CBM. The unstrained dielectric shows an isotropy in the energy range from 0 to 3 eV. However, the compressive and tensile strain make the optical spectrum of Kesterite CZGS/Se more anisotropic, specifically in terms of their low energy. The compressive enhanced the spectrum of polarization along the ZZ direction, which improved material absorption, opposite to the tensile strain that magnifies the peaks in polarized light along XX. The static value of the dielectric constant ($\varepsilon_{0}$) is calculated as the average value of ε(ω) at ω=0. The obtained value of $\varepsilon_{r}$ is 6.99 and 6.3 for CZGSe and CZGS, respectively. Under biaxial strain, the static constant along ZZ is increased from 6.31 to 8.31 by compressing the structure, from +6% to −6%. Meanwhile, $\varepsilon_{0}$ along XX decreases under both strains, following the same change in bandgap energy.

Figure 9c–f presents the change in $\varepsilon_{im}$ with a different biaxial strain applied. Similarly to the real part of ε, the imaginary part shows a high anisotropy with a higher strain percent. The compressive and tensile strain present a different effect in each direction of polarization. For the case of polarization along XX, the maximum of $\varepsilon_{im}$ increases with compression and decreases under tensile strain, contrary to the other direction, along ZZ, which shows the opposite effect.

**Figure 10:**
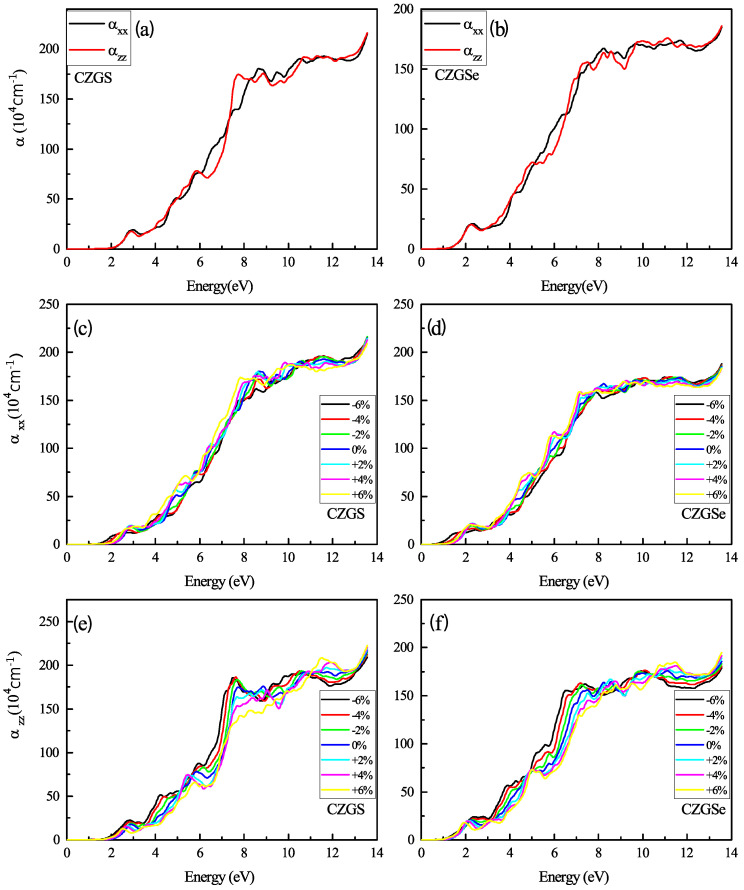
The variation of the absorption coefficient spectra as a function of the incident photon energy for both materials CZGS and CZGSe: (**a**,**b**) are the unstrained cases, (**c**–**f**) are the strained cases. $\alpha_{xx}$ and $\alpha_{zz}$ are the absorption coefficients for light polarization along XX and ZZ directions respectively.

**Figure 11:**
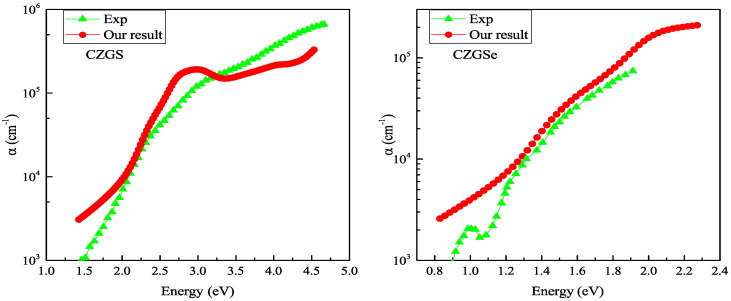
Experimental measurements of the optical absorption coefficient of CZGS [43] (**left**) and CZGSe [25] (**right**) are compared with those calculated from DFT + U (approach).

Figure 10 reports the absorption coefficient of both CZGS and CZGSe compounds for different considered biaxial strain rates. As can be seen in all cases, the materials present a high absorption coefficient of about 10${}^{4}$ cm${}^{-1}$ in the visible spectrum, which can make them suitable materials for thin films. For the strained cases (c–f), a slight shift can be observed in the positions of the peaks, but no significant differences were observed in the absorption magnitude. Despite the scarcity of experimental absorption measurements, which cover a wide range of energies, we tried to compare our results with some experimental data. Figure 11 shows that our theoretical predictions are in good agreement with the experimental data for both CZGS and CZGSe.

**Figure 12:**
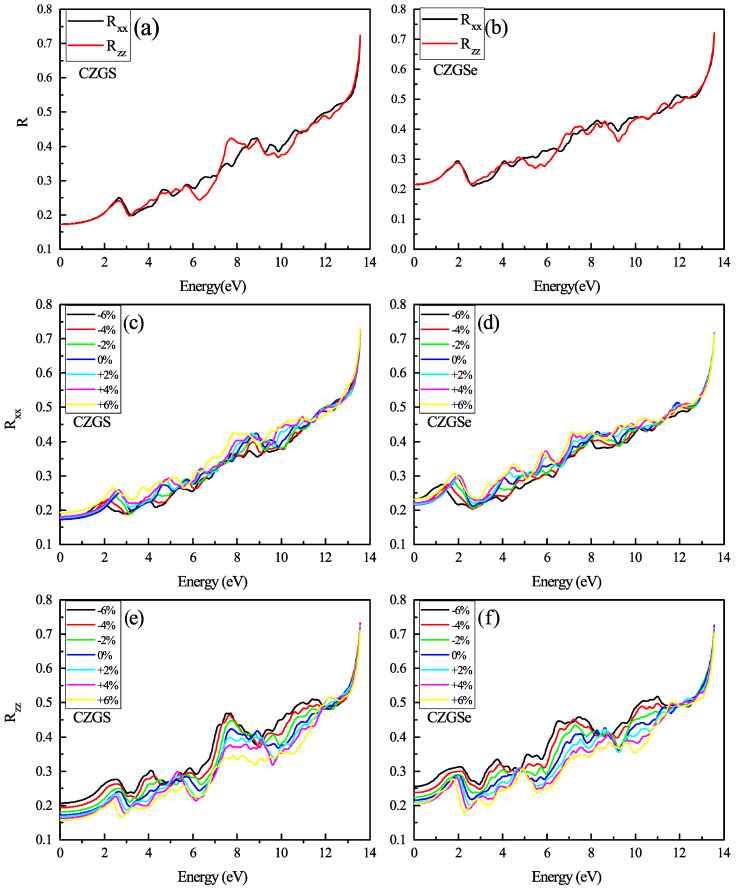
The variation of the reflectivity spectra as a function of the incident photon energy for both materials CZGS and CZGSe: (**a**,**b**) are the unstrained cases, (**c**–**f**) are the strained cases. $R_{xx}$ and $R_{zz}$ are the reflectivities for light polarization along XX and ZZ directions respectively.

The reflectivity of the unstrained CZGS and CZGSe is plotted in Figure 12a,b. Both materials present a low reflectivity for a low energy. The reflectivity increases with energy and the spectrum presents some peaks that correspond to the interband transition provoked by photon energy. We present the strained spectral of reflectivity $R_{XX}$ and $R_{ZZ}$ with a different strain percent in Figure 12c–f. Between the two directions of light polarization, the perpendicular reflectivity $R_{XX}$ is slightly affected by biaxial strain, steadily maintained its isotropy at a low energy; meanwhile, the parallel reflectivity $R_{ZZ}$ shows a high anisotropy in all the energy ranges from 0 to 13 eV. We observe that the compressive strain considerably increased the reflectivity of CZGS/Se, especially $R_{ZZ}$. The reflectivity values of unstrained CZGS/Se at 0 eV are $R_{XX}^{0\%}\left(0\right)=R_{ZZ}^{0\%}\left(0\right)=0.21$ for CZGSe and $R_{XX}^{6\%}\left(0\right)=0.18,R_{ZZ}^{6\%}\left(0\right)=0.16$ for CZGS; after applying compressive strain, the $R_{ZZ}$ value is raised to 0.2 and 0.25 for CZGS and CZGSe, respectively.

**Figure 13:**
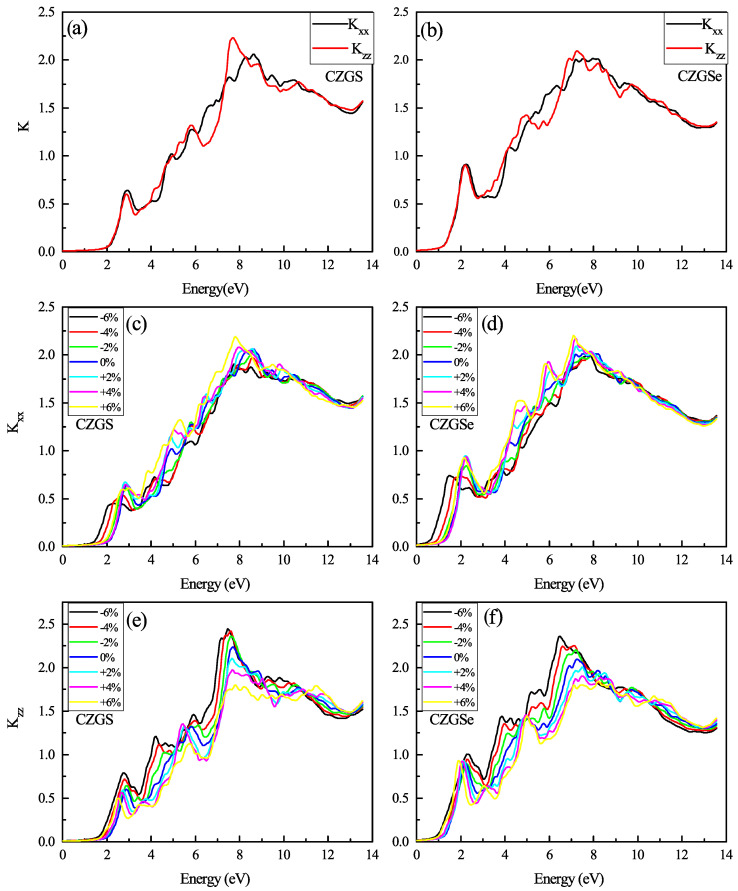
The variation of the extinction coefficient spectra as a function of the incident photon energy for both materials CZGS and CZGSe: (**a**,**b**) are the unstrained cases, (**c**–**f**) are the strained cases. $K_{xx}$ and $K_{zz}$ are the extinction coefficient for light polarization along XX and ZZ directions respectively.

Another optical property of Kesterite CZGS is the refraction index and extinction coefficient, which are the real and imaginary parts of the complex refraction index (N=n+ik) calculated from complex dielectric function parts. The spectrum of extinction coefficient in Figure 13a,b consists of many peaks related to transitions between the different occupied states in the valence band and the unoccupied state in the conduction band. The threshold energy of k for CZGS and CZGSe is equal to its band gap energy. After this, energy k increases, to reach its maximal value near 7 eV as an average between both polarizations. The subsequent extinction coefficient decreases considerably in both polarizations, which leads to a huge loss in the absorbed energy. The biaxial strain effect on the extinction coefficient is shown in Figure 13c–f. It is clear from the figure that lattice deformation shifts the threshold energy of k(ω) and the first peak toward lower energy. Generally, the peak intensity of perpendicular polarization deceases by compressing the structure; in contrast, it increases in parallel polarizarion to its maximum value of near 2 eV for both materials.

**Figure 14:**
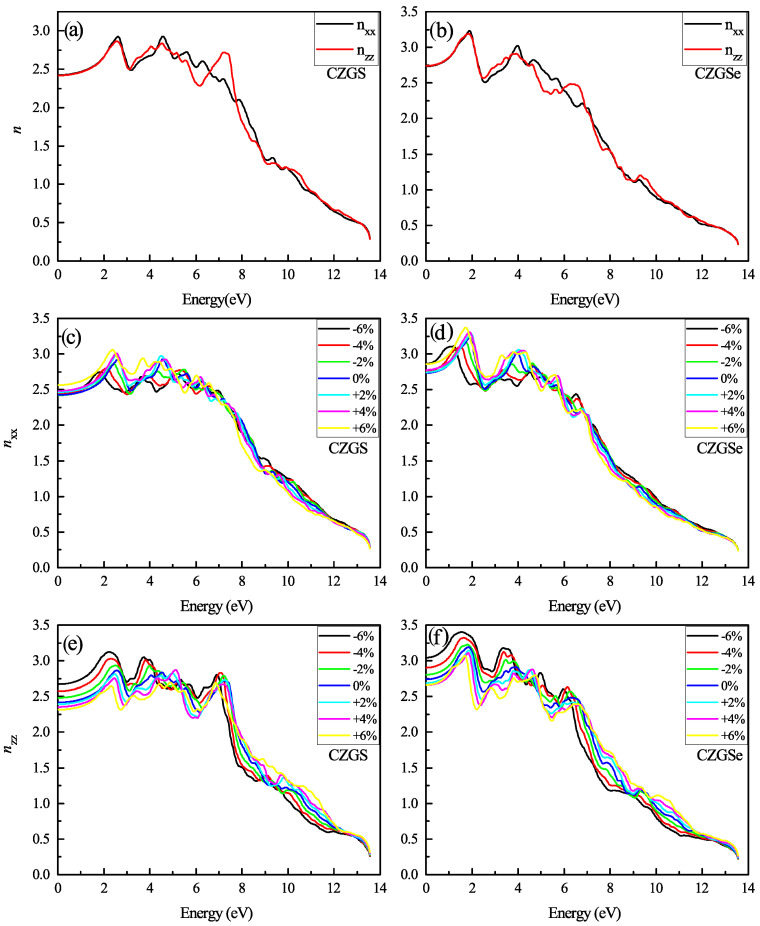
The variation of the refraction index spectra as a function of the incident photon energy for both materials CZGS and CZGSe: (**a**,**b**) are the unstrained cases, (**c**–**f**) are the strained cases. $R_{xx}$ and $R_{zz}$ are the refraction index for light polarization along XX and ZZ directions respectively.

**Figure 15:**
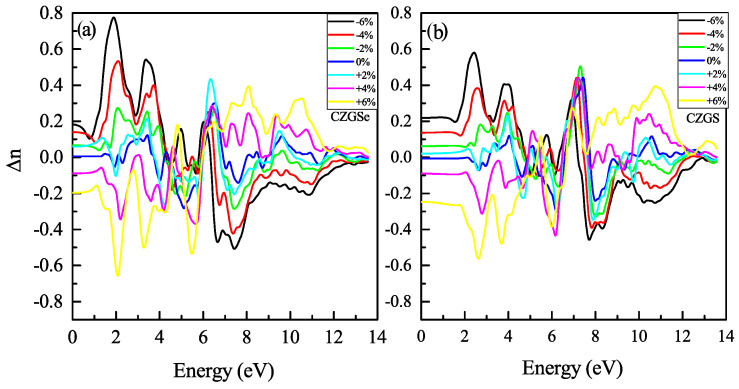
Spectral behavior of the birefringence for CZGS and CZGSe.

The refraction index is plotted in Figure 14a,b. The static refraction index for CZGS and CZGSe is 4.7 and 4.6, respectively. The refraction index increases to it maximum value near 2 eV for both materials. The strain induces the high anisotropy of the optical properties. After straining CZGS/Se, double refraction is observed, which leads to birefringence behavior in the materials. We present, in Figure 15, the spectral behavior of the birefringence Δn(ω) for the Kesterite CZGS/Se with different strain percentages. It is important to quantify the maximum difference between refractive indices exhibited by the material. Generally, birefringence Δn is defined as the difference between the extraordinary and ordinary refraction indices, $\Delta n=n_{e}-n_{o}$, where n${}_{e}$ and n${}_{o}$ are the extraordinary and ordinary refraction indices [49], for the kesterite materials, which, respectively, represent the parallel and perpendicular refraction index of CZGS/Se. It is well known that the material has a positive uniaxial birefringence if its birefringence Δn(0) is positive, and it is said that the materials with a negative Δn(0) have negative uniaxial birefringence. As can be seen from Figure 15, the birefringence is found to be more remarkable in two significant regions. The first region corresponds to the range of energy from which the absorption coefficient is an increasing function. The birefringence is positive for the compressive strain and negative for the tensile one for both materials. The second region represents the region of high energies, in which the absorption stagnated (see Figure 10), where the birefringence behaviour was reversed. We note that CZGSe presents a higher birefringence behaviour than CZGS. These changes in the optical properties are mainly due to the break of symmetry induced by straining the structure.

## 4. Conclusions

DFT calculations of strain effect on the electronic and optical properties of Cu${}_{2}$ZnGeS${}_{4}$ and Cu${}_{2}$ZnGeSe${}_{4}$ in their kesterite structure were performed using GGA, mBJ, and U exchange-correlation potentials. We have provided further evidence that the strain has a remarkable influence on both electronic and optical properties. The results demonstrate that the band gap decreases from 2.05 and 1.26 for the unstrained structures CZGS and CZGSe to values around 1.5 and 0.7 eV in a compressed structure, while the values are around 1.7 and 0.9 eV for a stretched structure. The band gap energy decreased with strain, giving more opportunities to this type of kesterite to become very widespread in solar cell manufacturing. The dielectric functions presented a high anisotropy in their spectrum, as well as the strain, which becomes more pronounced. The related absorption coefficient, reflectivity, extinction coefficient, and refraction index were calculated as functions of applied biaxial strain. A significant anisotropy is found in the spectra of all these optical coefficients.

## Figures and Tables

**Table 1:** Calculated lattice constants a and c, band gap energy E${}_{g}$, static dielectric constant $\varepsilon_{0}$, and refraction index $n_{0}$ by DFT/PBE + (mBJ + U) approach for CZGS and CZGSe quaternary semiconductor compounds.

	CZGS			CZGSe		
		Th	Exp		Th	Exp
a (Å)	5.28	5.26 ${}^{c}$	5.27 ${}^{c}$	5.58	5.602	5.59
		5.35 ${}^{b}$	5.34 ${}^{d}$		5.71	–
c (Å)	10.51	10.84 ${}^{c}$	10.50 ${}^{c}$	11.11	11.25 *^e^*	11.04 ${}^{f}$
		10.64 ${}^{b}$	10.51 ${}^{d}$		11.27 ${}^{a}$	–
E${}_{g}$ (eV)	2.05	0.76 ${}^{c}$	1.88–2.23 ${}^{f}$	1.28	0.64 *^e^*	1.17–1.52 ${}^{g}$
		2.14 ${}^{f}$	–		1.6 ${}^{c}$	–
$\varepsilon_{0}$	5.88	6 ${}^{a}$	–	7.47	7.36 ${}^{a}$	–
$n_{0}$	2.42	2.45 ${}^{a}$	–	2.73	2.71 ${}^{a}$	–

${}^{a}$ [34]. ${}^{b}$ [35]. ${}^{c}$ [45]. ${}^{d}$ [46]. *^e^* [47]. ${}^{f}$ [27]. ${}^{g}$ [25].

## Data Availability

The data will be able on request.
